# Detection and Characterisation of Wasp Venom‐Specific T Cells Using the ARTE Method in Allergic Patients

**DOI:** 10.1111/cea.14606

**Published:** 2024-12-15

**Authors:** Magdalena Kraft, Samira Saleh, Guido Heine, Alexander Scheffold, Petra Bacher, Margitta Worm

**Affiliations:** ^1^ Department of Dermatology, Venereology and Allergology Charité—Universitätsmedizin Berlin Berlin Germany; ^2^ Department of Dermatology and Allergy University Hospital Schleswig‐Holstein, Campus Kiel Kiel Germany; ^3^ Institute of Immunology Christian‐Albrechts‐University of Kiel and University Medical Center Schleswig‐Holstein Kiel Germany; ^4^ Institute of Clinical Molecular Biology Christian‐Albrechts‐University of Kiel and University Medical Center Schleswig‐Holstein Kiel Germany

**Keywords:** allergy, anaphylaxis, antigen‐specific T cells, ARTE, IgE, Th2 cells, venom

**Summary**

- Venom‐specific T cells can be detected in peripheral blood after initiation of VIT with ARTE.
- These cells showed a memory phenotype and produced IL‐4 but not IFNγ.

**Abbreviations**
ARTE: antigen‐reactive T cell enrichment
IFNγ: interferon γ
IgE: immunoglobulin E
IgG: immunoglobulin G
IL: interleukin
sIgE: specific immunoglobulin E
Tfh: T follicular helper cell
Th: T helper cell
VIT: venom immunotherapy

To the Editor,

Hymenoptera venom is one of the major elicitors of anaphylaxis. These reactions are mediated by specific immunoglobulin E (sIgE) that results from B‐ and T‐cell interactions, where antigen‐specific T cells provide crucial survival, proliferation and class‐switch promoting factors to B cells. The presence of venom sIgE and sIgG and their kinetics after a sting and during immunotherapy in individuals with venom allergy have been well described. Conversely, venom‐specific T cells have rarely been investigated because of the difficulties in detecting them in the peripheral blood. Previous studies have mainly used indirect methods, such as in vitro cultures or peripheral blood mononuclear cell (PBMC) stimulation with venom and cytokine measurement [1, 2, 3, 4]. These methods enabled the initial characterisation of venom‐specific T cells and their alterations during venom immunotherapy (VIT). However, they do not enable the evaluation of multiple characteristics of polyclonal antigen‐specific T‐cell responses at the single‐cell level in various patient groups.

Here, we describe the first analysis of venom‐specific T cells using the Antigen‐reactive T cell enrichment (ARTE) method [5]. For details, see Methods in the online supplement: https://osf.io/cwd52/. Briefly, PBMCs from patients with confirmed wasp allergy were collected and analysed before initiating VIT and 1 week after the rush induction of VIT. At baseline, venom‐specific T cells were detectable only in some patients (7/19; 37%, Figure 1A). In contrast, they were detectable in most patients (17/19; 89%) 1 week after rush VIT initiation. The frequencies of antigen‐specific cells against *Candida albicans* antigen MP65 (used as a positive control here, as T cells reacting to this antigen are almost always detectable at a steady state) did not change between both time points (Figure 1A). The cytokine expression profiles against both antigens differed significantly: We observed IL‐4, IL‐13 and IL‐5 expression among wasp venom‐specific T cells (Figure 1B), indicating that some of them were Th2 or Tfh cells. Candida‐specific T cells did not express Th2 cytokines. Conversely, they showed a higher expression of IL‐2 and IFNγ (Figure 1B). The expression of any of the cytokines did not change significantly with VIT induction, neither among venom‐specific nor *Candida*‐specific T cells (data not shown). Nearly all detected venom‐specific T cells had a CD45RO+ memory phenotype comparable to *Candida*‐specific cells (Figure 1B). The frequency/detection rate of venom‐specific T cells and their cytokine production profiles were independent of the patient's age, sex or the severity of previous anaphylactic reactions and not correlated with tryptase levels (data not shown). However, venom‐specific T cell frequencies correlated with total IgE, and with venom‐specific sIgE (data not shown). Venom sIgE also correlated positively with the proportion of IL‐4‐producing venom‐specific T cells (data not shown).

**FIGURE 1:**
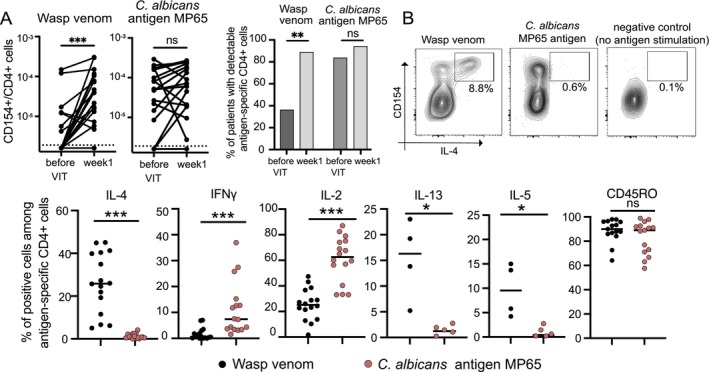
Wasp venom‐specific IL‐4, IL‐13 and IL‐5‐producing memory T cells can be detected in the blood of allergic donors. (A) Frequencies of venom or MP65 reactive CD154+ T cells among CD4+ PBMCs from wasp venom allergic donors and per cent of patients with detectable levels of these cells before and 1 week after initiation of VIT. Only patients where data regarding both antigens at both time points was available were included in the analysis (*n* = 19). Values below the detection limit were plotted on the *x*‐axis under the dotted line for graphical presentation purposes. The frequencies of antigen‐specific T cells were compared using the Wilcoxon test for paired samples, and the percentages of patients with detectable antigen‐specific T cells using Fisher's exact test. ***p < 0.001, **p < 0.01, ns—not significant. (B) Representative example staining for IL4+ CD154+ of CD4+ PBMCs (predated on single live, CD14−, CD20− and CD8− CD4+ lymphocytes) for venom (left panel) positive control ( *Candida albicans* MP65 antigen; middle panel) and negative control (no antigen during stimulation; right panel) after ARTE enrichment 1 week after initiation of VIT as well as the quantification (percentage) of IL4+, IFNγ+, IL‐2+, IL‐13+ or IL‐5+ or CD45RO+ cells among CD154+ CD4+ PBMCs after stimulation with wasp venom or *C. albicans* MP65 antigen 1 week after initiation of VIT. Only samples with detectable venom or MP65 antigen‐reactive CD154+ cells were included in the analysis. The line represents the median. Mann–Whitney test. ***p < 0.001, *p < 0.05, ns—not significant.

The estimated frequencies of the venom‐specific T cells among all CD4+ cells measured with ARTE, despite the antigen rechallenge (initiation of VIT), were 1.5–2.5 log lower than that of common pathogens, such as *C. albicans* or cytomegalovirus [5], or common aeroallergens [6]. Interestingly, we detected no venom‐specific T cells in most patients before starting VIT, although they were detectable after VIT initiation. Conversely, allergen‐specific T cells against aero‐ and food allergens can be detected in individuals not undergoing SIT [6, 7, 8]. This might be due to the limitations of our method (e.g., limitations in venom‐antigen presentation ex vivo and the composition of the extract used for the stimulation, or too conservative threshold for limit of detection), but this might also reflect the necessity of continuous exposure via the skin or mucosa and restimulation of antigen‐specific T cells which then circulate in the peripheral blood at detectable frequencies. As most venom‐specific T cells detected upon VIT were CD45RO+, the increasing frequencies reflected a clonal expansion and not de novo induction of these cells during VIT.

Our results indicate that a significant fraction of venom‐specific T cells express IL‐4, some of which express IL‐5 and IL‐13. Interestingly, the fraction of IL‐4‐expressing cells in our study was similar to that reported for food or aeroallergens [6, 8]. However, the fraction of IL‐5‐expressing cells was approximately half lower, which might indicate that cells with an enhanced type 2 profile (Th2+/Th2A/Tfh2) are less abundant among venom‐specific T cells than among other allergen‐specific T cells. We detected only very few IFNγ‐producing venom‐specific T cells, which confirms some of the previous studies, where IFNγ production from T cells after stimulation with venom was detectable only after at least 2 months of VIT, but not before [3]. Conversely, Subramaniam et al. [4] reported IFNγ‐producing CD1a‐reactive T cells in venom‐allergic individuals, which were 2–5 times more frequent than the IL‐13‐producing cells detected using ELISpot. Further studies are required to investigate whether the methods employed (flow cytometry vs. ELISpot) caused this discrepancy or whether CD1a‐reactive T cells respond to challenges with venom lipids and endogenous lipids generated by venom with increased IFNγ and decreased IL‐13 production compared with peptide‐specific T cells. We observed that approximately 25% of venom‐specific T cells produced IL‐2, corresponding to a similar fraction of IL‐2‐producing peanut‐specific T cells reported as recently published [9].

Altogether, our data revealed the presence of venom‐specific T cells in patients with venom allergies, including memory Th2/Tfh2 cells, which produce IL‐4, IL‐5 and IL‐13 but not IFNγ. Therefore, we conclude that ARTE is a suitable method to detect and characterise venom‐specific T cells with high precision, thereby helping to understand the basic immune processes underlying antigen‐specific immunotherapy against venom allergy.

## Author Contributions

M.W., G.H., A.S., and P.B. designed the study. S.S. carried out the flow cytometry experiments. M.K. analysed the data and wrote the manuscript. All co‐authors contributed to the interpretation of the results and writing of the final manuscript.

## Conflicts of Interest

MK reports travel support from ALK‐Abéllo and speaker honoraria from Bencard Allergie GmbH, outside the submitted work. MW received reimbursement for speaker and/or consulting activities from Novartis Pharma GmbH, Sanofi‐Aventis Deutschland GmbH, DBV Technologies S.A, Aimmune Therapeutics UK Limited, Regeneron Pharmaceuticals Inc., Leo Pharma GmbH, Boehringer Ingelheim Pharma GmbH & Co. KG, ALK‐Abelló Arzneimittel GmbH, Lilly Deutschland GmbH, Kymab Limited, Amgen GmbH, AbbVie Deutschland GmbH & Co. KG, Pfizer Pharma GmbH, Mylan Germany GmbH (A Viatris Company), AstraZeneca GmbH, Lilly Deutschland GmbH, GlaxoSmithKline GmbH & Co. KG and Almirall Hermal GmbH. GH reports grants from Deutsche Forschungsgemeinschaft (DFG), during the conduct of the study; personal fees from Allergopharma and speaker Honoria from Abbvie, ALK Abelló, Biotest, Eli Lilly, Leti and Sanofi, outside the submitted work. The rest of the authors declare no relevant conflicts of interest.

## Data Availability

The data that support the findings of this study are available on request from the corresponding author. The data are not publicly available due to privacy or ethical restrictions.
